# Force of Infection (FOI) and Multiplicity of Infection (MOI) in *Plasmodium falciparum* Infected Children Aged 1.5–12 Years Living in the Malaria Endemic Area of Banfora, Burkina Faso

**DOI:** 10.3390/pathogens13100883

**Published:** 2024-10-10

**Authors:** Emilie S. Badoum, Ludovic Kouraogo, Amidou Diarra, Daouda Ouattara, Issa Nebie, Alphonse Ouedraogo, Alfred B. Tiono, Sodiomon B. Sirima

**Affiliations:** Groupe de Recherche Action en Santé (GRAS), Ouagadougou 06 BP 10248, Burkina Faso; ludovickouraogo94@gmail.com (L.K.); a.diarra@gras.bf (A.D.); d.ouattara@gras.bf (D.O.); i.ouedraogo@gras.bf (I.N.); a.ouedraogo@gras.bf (A.O.); a.tiono@gras.bf (A.B.T.); s.sirima@gras.bf (S.B.S.)

**Keywords:** infection, malaria, molecular force, multiplicity, *msp2*, *P. falciparum*, polymorphism

## Abstract

The aim of this study was to explore molecular measures of *P. falciparum* malaria burden (FOI and MOI) in the context of seasonal malaria chemoprevention. We analyzed malaria cases collected as part of a longitudinal cohort study. The cohort included *P. falciparum*-negative children aged 1.5 to 12, as confirmed by PCR 21 days after a radical cure using DHA-PQ or AS. Children were followed up for six months using active and passive case detection methods. At each visit, dried blood spots and blood smears were collected by finger prick, along with clinical data. Parasite DNA was extracted and analyzed by nested PCR for detection and genotyping of *P. falciparum* parasites. A total of 458 *P. falciparum* isolates collected during follow-up from October 2020 to March 2021 were genotyped. During the follow-up, children contracted 1.05 (95% IC [0.81–1.30]) new *P. falciparum* infections/child/time of exposure, and the MOI value was 3.00 (SD 1.60). Age is a protective factor (IRR: 0.74; 95% CI: 0.61, 0.90) against the occurrence of an episode of malaria, unlike an increase in MOI (IRR: 1.63; 95% CI: 1.04, 1.99), which is a favorable factor (*p* < 0.05). This study confirms the reduction in malaria transmission in our study area, probably due to the massive deployment of control tools.

## 1. Introduction

According to World Health Organization (WHO) estimates, there were 247 million cases of malaria worldwide in 2021 compared to 249 million in 2022, with most of this increase coming from countries in the WHO’s African Region. Twenty-nine countries accounted for 95% of global malaria cases, and four African countries accounted for almost half of all cases globally. The WHO African Region, with an estimated 233 million cases in 2022, accounted for about 94% of cases and just over half of all malaria deaths worldwide [1].

In Burkina Faso, malaria is a major public health problem. Data from WHO on the malaria burden in 2020 estimated more than 10 million suspected malaria cases and roughly 4000 deaths [1]. To address this problem, Burkina Faso has subscribed to several global initiatives such as the “Roll Back Malaria” initiative to fight the disease. To this end, several strategies have been implemented, the most important of which are: the adoption in 2005 of Artemisinin-based Combination Therapies as first-line treatment for uncomplicated malaria, the adoption of Intermittent Preventive Treatment with Sulfadoxine-Pyrimethamine for pregnant women [2,3], and the Chemoprevention of Seasonal Malaria in children aged 3 to 59 months in 2014 [4], as well as the massive distribution of long-lasting impregnated bed-nets.

Despite these malaria control strategies, the resistance of the parasite (*Plasmodium*) to antimalarial drugs adds to the epidemiological and economic burden of the scourge in many endemic countries [5]. Various factors, in particular the diversity and antigenic variation of the parasite responsible for the slow acquisition (several years) of protection against malaria, make malaria control complex [6].

Genetic diversity of malaria parasites is essential in understanding the mechanism underlying malaria pathology and determining parasite clones’ profiles in an infection for proper malaria control strategies such as malaria vaccine development, a crucial tool for malaria eradication. Indeed, the malaria vaccine development is complicated by the high genetic diversity of the parasite populations, which impede the setting of protective acquired immunity against malaria [7]. This genetic diversity within *P. falciparum* is a fundamental propriety by which the parasites escape the hosts’ immune responses, and it results from allelic polymorphism, recombination, chromosome rearrangements, and antigenic variation [8]. The mechanisms controlling this diversity within the parasite genome are many and complex. In several studies, according to malaria transmission intensity, individuals are often infected simultaneously by several parasite clones [9,10,11]. The described extensive polymorphisms are related to some key parasite genes coding antigens during the parasite’s asexual blood stage [12]. Indeed, polymorphisms of blocks 2 and 3 of the gene coding for the MSP1, MSP2, and GLURP, respectively, of *P. falciparum* have long been used as genetic markers for genotyping of parasite populations [13]. Several studies have demonstrated that the locus of the *msp2* gene of *P. falciparum* is extremely polymorphic [14] and, therefore, most informative.

A previous study has shown that, for a better measurement of the outcomes of malaria control interventions, genotyping of *P. falciparum* parasites in longitudinal studies provides a robust approach to estimating the genomic metrics of transmission [13]. Two genetic parameters have been proposed for study of the effects of these interventions at the molecular level: the force of infection (FOI) and the multiplicity of infection (MOI). The FOI, defined as the number of new *Plasmodium* clone infections acquired over time [13], is an alternative measurement of malaria transmission. The multiplicity of infection (MOI) is defined as the number of simultaneous parasite clones per *P. falciparum*-positive host [13]. In endemic areas, multiple infections of *P. falciparum* clones are common, and the MOI varies with the degree of malaria endemicity [14].

At a potential vaccine trial site (southwest Burkina Faso, where malaria is endemic and seasonal), we performed a molecular characterization of highly polymorphic genetic MSP2 of *P. falciparum* malaria to explore the FOI and MOI with their influencing factors, which are valuable to inform the design of malaria vaccine trials.

## 2. Materials and Methods

### 2.1. Study Site

**Figure 1:**
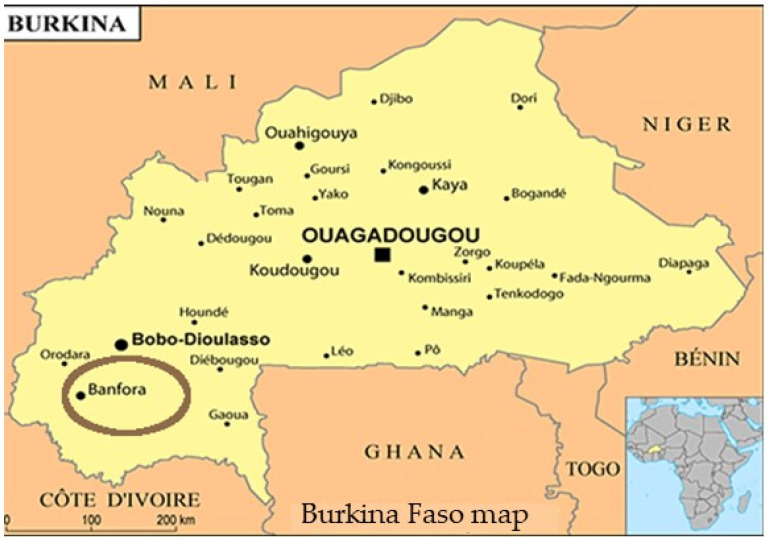
Map of Burkina Faso giving Banfora location (https://jumelage-pessac.org/ville/banfora) (accessed on 28 September 2024).

The study was conducted in an area covered by the Banfora Health District, located in the southwestern part of Burkina Faso (Figure 1). The population in the Cascades region was estimated in 2019 at 812,466 inhabitants, among whom 633,043 are residents of the administrative province of the Comoé [15]. This district covers a surface area of 15,405 km^2^. The Banfora Health District has intense seasonal malaria transmission over a six-month period following seasonal rains from May to November [16]. *P. falciparum* accounts for >90% of malaria cases [16]. With the WHO’s recommendation to introduce Seasonal Malaria Chemoprevention (SMC) since 2012 [17], children under 5 years in the study area have benefited from this intervention during the high-transmission season since 2014, as has the rest of the country [4,17].

### 2.2. Study Population, Design and Period

Data for this study were obtained from a longitudinal cohort study, aiming primarily to assess the incidence of clinical malaria in children aged 1.5 to 12 and to identify the most effective antimalarial drug to use for a pre-vaccination radical cure strategy. Male or female children aged 1.5 to 12 years and living permanently in the study area were included in the cohort. They were malaria-free as confirmed by a negative PCR 21 days after a malaria radical cure using either artesunate (AS) alone or dihydroartemisinin-piperaquine (DHA-PQ), and their parents or guardians provided written informed consent. Children were followed for up to six months from October 2020 to April 2021 using both active and passive case detection methods to ensure the capture of a high proportion of malaria infections in the cohort. The active case detection consisted of fortnightly visits to the child at home. During each visit, clinical data were collected; then, malaria smears and dried blood spots were prepared using a finger prick. Between two active visits, parents were encouraged to take their child to the local health facility whenever he/she felt unwell. During these passive visits, the same procedures as for the active visits were conducted. We took advantage of this cohort study to describe *P. falciparum* genetic diversity, targeting the most polymorphic region of *msp2* gene. All the malaria infection cases that occurred during the active and passive follow-up periods were used to characterize *msp2* allelic families. In addition to the MOI, the longitudinal cohort follow-up was appropriate to estimate the FOI.

### 2.3. Sample Collection

Dried blood spots on Whatman filter papers (Sigma, Pittsburg, PA, USA) were labeled, then air-dried and placed individually in plastic bags containing a desiccant, thus protecting them from humidity. These filter papers were used for the analysis of *P. falciparum*’s presence by PCR. Thick and thin blood smears were prepared on the slide for malaria detection by microscopy. Samples were collected through a finger prick.

### 2.4. Malaria Parasites Density by Microscopy

Thick and thin blood films were air-dried and stained with 6% Giemsa. Each slide was read by two laboratory technicians. Asexual and sexual parasites were counted separately and species differentiated. Malaria parasites were counted against 200 white blood cells (WBC). A slide was declared negative only after reading against 100 microscopic fields without observation of a malaria parasite. Parasite densities were calculated assuming an average of 8000 white blood cells/µL of blood. The arithmetic mean of the two readings was used as the final parasite density. In the event of a discrepancy over the presence or absence of malaria parasites between the two readers, or if parasite density estimates differed by more than 30%, the slide was re-examined by a third laboratory technician. The arithmetic mean of the two most concordant results (out of the three readings) was considered as the final parasite density.

### 2.5. DNA Extraction by Methanol Method

DNA was extracted using the ethanol method. For that, three pieces of filter paper were soaked in methanol (100 µL) for 15 min at room temperature. After 15 min of action, the methanol was transferred from the tube and the pieces were left to dry completely under vacuum or in the open air for 1–2 h. After drying, 100 µL of sterile distilled water was added to each tube. The tubes were then heated in a water bath for 15 min at 95–100 °C. During the incubation phase, the tube was vortexed every 5 min to extract plasmodial DNA. The DNA extracts were used directly.

### 2.6. Molecular Analysis

The *P. falciparum* species was identified by a nested PCR amplification from two PCR reactions. This is a nested PCR method targeting the 18 s rRNA small sub-unit gene. Products obtained after the first PCR were amplified using specific primers for *P. falciparum*. The sequence of the primers and the protocol of PCR are described in detail elsewhere [18]. Primary and secondary PCRs were carried out in a final volume of 20 μL containing 4 μL of Master Mix (5x FIREPol^®^, 1.25 μL of dNTP, 0.8 μL of 25 mM MgCl_2_, 0.1 μL of One Taq^®^ DNA polymerase) and 1 μL of Template DNA or primary PCR product under the following conditions: 95 °C for 5 min, 58 °C for 2 min, and 72 °C for 2 min, followed by cycles (24 for primary PCR and 30 cycles for secondary PCR) of 94° C for 1 min, 58 °C for 2 min, and 72 °C for 5 min. After amplification, samples were subjected to electrophoretic migration on a 1.5% agarose (Sigma Aldrich Chemie GMBH, Taufkirchen, Germany) gel and 3% GelRed^®^ (Nucleic Acid Stain) in Tris Borate Ethylene-Diamine-Tetra-Acetic. Migration was performed at 100 V for 90 min. Fragment size was determined using the molecular weight marker. UV electrophoresis development was carried out using an image documentation system (Axygen, Corning, NY, USA) coupled to a computer, enabling the exact size of DNA bands to be estimated. The expected fragment length was 205 base pairs (bp) for *P. falciparum* [18].

*msp2* (central region) was genotyped using a nested PCR. Briefly, products obtained after the first PCR were amplified using specific primers of the two distinct allelic families for *msp2* (FC27 and 3D7) in accordance with the recommended genotyping protocol [19]. The sequence of the primers and the protocol of PCR were previously described in detail by Snounou et al. [20]. Both primary and secondary PCRs for *msp2* were carried out in a final volume of 25 μL containing 4 μL of Master Mix (5x FIREPol^®^, 1.25 μL of dNTP, 0.8 μL of 25 mM MgCl_2_, 0.1 μL of One Taq^®^ DNA polymerase) and 1 μL of Template DNA or primary PCR product under the following conditions for primary and secondary PCR (30 cycles): initial denaturation at 95 °C for 5 min, extension at 94 °C for 1 min, annealing at 58 °C for 2 min for the primary and 61 °C for 2 min for the secondary amplification, extension at 72 °C for 2 min, and final elongation at 72 °C for 10 min for the primary and 72 °C for 5 min for the secondary amplification. All PCRs were performed in an Applied Biosystems 2720 thermal cycler (Applied Biosystems, Walthman, MA, USA). As with *P. falciparum* identification, the band lengths of the different allelic families were detected after migration on an agarose gel. Using the molecular weight marker (100 bp), we determined the lengths of the different fragments.

### 2.7. Data Management and Statistical Analysis

Data were directly recorded on android tablets, double-checked by the investigators, and transferred to the database. Controls and edit checks were included in the data capture system to ensure data quality.

The mean MOI was calculated by dividing the total number of alleles detected by the total number of positive samples. The FOI was calculated from the number of new *P. falciparum* infections, expressed as the number of new infections per unit time, and determined by counting all new msp2 genotypes not present in preceding visits [13]. Multiclonal infections were defined as infections with more than one allele of the *msp2* gene. Molecular data were generated and recorded on an Excel sheet, checked for consistency, and merged with the rest of the dataset. Data were cleaned before being exported for analysis using R version 3.5.1. Descriptive statistics (means and proportions) were calculated with their 95% confidence intervals. As part of the analysis of the different predictive factors (a model with several independent variables), a multivariate analysis was carried out to assess their impact. A clinical malaria episode was defined as objective fever (axillary temperature ≥ 37.5 °C/tympanic ≥ 38 °C or forehead temperature ≥ 37.5 °C using a non-contact infrared thermometer (Microlife, Ängelholm, Sweden)) and parasitemia of >2500 parasites/µL by microscopy.

The Pearson chi-square test was used for the comparison of proportions and frequencies or the Fisher exact test for the comparison of proportions when the theoretical number was less than 5. The Student test was used for comparison of means. The *p*-values were reported, with differences considered significant at *p* < 0.05.

## 3. Results

### 3.1. Baseline Characteristics of Participants

**Figure 2:**
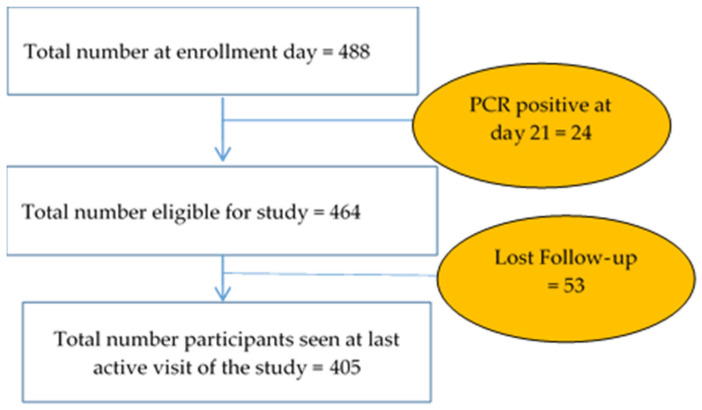
Follow-up profile.

For a total of 488 children enrolled, 464 (95.08%) were PCR-negative on Day 21 after anti-malarial treatment for radical cure. A total of 405 (87.28%) subjects completed the study (Figure 2).

Children aged 1.5 to 5 years accounted for 41.16% (191) of the sample, and 58.84% (273) were older (aged 5 to 12 years). Both genders were equally represented, with 229 (49.35%) males and 235 (50.65%) females. As shown in Table 1, insecticide-treated mosquito nets (ITN) alone as a means of protection were used by less than a quarter of the study population (20.30%). The combination of insecticide-treated mosquito nets and other malaria prevention methods (mosquito repellent cream or spray, coils, traditional or herbal preventive methods) was more widely used (77.32%). However, there were patients who did not use any method of protection.

### 3.2. Incidence of P. falciparum Infection by PCR during the Study

A 6-month follow-up was carried out, combining active (with a bi-weekly sampling) and passive surveillance. Thus, the cumulative incidence of *P. falciparum* infection was 44.11% [39.55–48.76], with a significant difference (*p* = 0.049) according to age (38.32% [31.40–45.73] for subjects under five years and 48.01% [42.02–54.06] for the elder group).

### 3.3. FOI and MOI in Study Participants

This study successfully identified *msp2* allelic families (3D7 and FC27) in 87.99% of *P. falciparum* isolates, with the 3D7 family (82.63%) exhibiting a significantly higher frequency (*p* = 0.001) compared to the FC27 family (73.20%). The MOI value was 3.00 (SD 1.60), with a slight decrease with age (3.15 (SD 1.67) for subjects aged 1.5 to 5 years and 2.95 (SD 1.58) for subjects aged over 5 years), but with no statistically significant difference (*p* = 0.16). The FOI was 1.05 (95% IC [0.81–1.30]) new *P. falciparum* infections per child per time of exposition. Subjects under 5 years had an FOI of 0.71 (95% IC [0.36–1.04]) new *P. falciparum* infections per child per time of exposition versus 1.28 (95% IC [0.94–1.62]) new *P. falciparum* infections per child per time of exposition for subjects over 5 years. This difference between age groups was statistically significant (*p* = 0.023).

The prevalence of multiple infections (polyclonality) was close to 80% in all cases. However, this parameter did not seem to be affected by age group (*p* = 0.53), with 84.44% for <5 years and 80.83% in children ≥5 years.

### 3.4. Risks Factors of Clinical Episode

In total, 21 children developed a clinical episode during the follow-up period. The results in Table 2 show that, with the unadjusted model, only MOI and age were significantly associated with the occurrence of clinical malaria episodes. The same observations were valid in the adjusted model.

### 3.5. Predictive Factors of MOI and FOI

#### 3.5.1. Predictive Factors of MOI

Multivariate analysis (Table 3) of different factors showed that only polyclonality (IRR: 2.962; 95% CI: [2.17–4.03), parasitemia (IRR: 1.239; 95% CI: [1.06–1.45]), FOI (IRR: 1.168; 95% CI: [1.12–1.22]), and rainy months (IRR: 1.680; 95% CI: [1.38–2.04) were predictive factors given the adjusted results (*p* ˂ 0.05).

#### 3.5.2. Predictive Factors of New Clones’ Acquisition (FOI)

The summary of our findings is presented in Table 4. In multivariate Poisson regression, pre-existing polyclonality (IRR: 2.46; 95% CI: [1.62–3.76]), MOI (IRR: 1.27; 95% CI: [1.22–1.34]), and study month (IRR: 2.09; 95% CI: [1.67–2.63]) were found to significantly influence the acquisition of new clones.

## 4. Discussion

In this study, we explored molecular measures of FOI and MOI using the *msp2* gene in children aged from 1.5 to 12 years after they had received a radical cure consisting of antimalarial drugs to eliminate existing *P. falciparum* parasites.

The high diversity of the *msp2* gene is due to an allele-specific core region that includes repeats of varying lengths. The alleles are grouped into two distinct families, the 3D7 family (Indochina) and the FC27 family [21,22], based on the dimorphic structure of the non-repeat variable region [21]. These features make *msp2* a suitable genetic marker for genotyping *P. falciparum* infections and provide an informational tool for enumerating multiple simultaneous infections in a blood sample and for distinguishing individual alleles [23,24]. Better knowledge of *P. falciparum* genetic diversity could improve our understanding of malaria’s pathological mechanisms, acquired immunity processes, spread, and the genetic background of drug resistance and transmission conditions [25].

An increase in the age of participants was associated with a reduced risk of occurrence of a clinical episode. This is in line with previous observations that acquisition of premunition increases with age and, therefore, older children are expected to be at a lower risk of malaria episodes. The results of previous studies have reported that school-aged children (aged over five years) are the main reservoir of malaria parasites [26,27,28]. In addition, the malaria control methods introduced in Burkina Faso targeting young children, especially under-fives, may have contributed to better protection of the younger children in this study.

The mean value of the MOI obtained in this study could be explained by the climatic and environmental conditions and the humidity of the area, which favor the maintenance of high and perennial transmission in this part of Burkina Faso. It was close to that found in 2020 in Burkina Faso [29], relatively higher than the values previously found in the country [30,31,32], and largely different from those found by Tadele et al. in Ethiopia in 2022 [33]. In our study, all participants received a radical cure to clear all *P. falciparum* parasites before their enrolment and were divided into two groups; the under-five-years subjects who were under SMC and the non-SMC over-five-years children had similar multiclonal infections. This would suggest that SMC did not alter the infection trend. In addition, studies have shown that when chemoprophylaxis is used, malaria may be atypical and those infected may have symptoms. Previous studies on the variation in MOI with age have suggested that this influence on multiplicity of infection may be strongly affected by malaria’s endemicity [34,35]. Studies have also shown that MOI is age-dependent in areas of intense and perennial malaria transmission, but not in areas of meso-endemic malaria [34,35]. A comparable trend of a positive association between multiplicity and age was observed in a previous study by Soulama et al. in Burkina Faso [31], but Arzika et al. [36] did not observe an age-dependent pattern in Niger.

A high prevalence of multiclonal infections was observed in this study, which could be due to high parasite pressure consecutive to continuous and intense malaria transmission, suggesting efficient genetic recombination of the parasite population within the female anopheles [37,38]. It could also result from multiple independent inoculations of single-parasite clones [39]. Simultaneous infections of a large number of individuals with several different parasite genotypes in areas of high transmission intensity have been attributed to multiple inoculations of single clones or single inoculation of multiple clones (co-transmission) that may have undergone crossover and recombination in the female anopheles [40,41]. In a study conducted by Soulama et al., the data showed that older subjects were more often infected by several different clones [28] in contrast to Sondo et al., where the MOI decreased significantly in older hosts [26]. Previous studies have suggested that high genetic diversity in the parasite population may lead to a progressive selection of more virulent strains, which in turn may lead to the emergence and proliferation of drug-resistant parasites [42,43].

The FOI is particularly useful as an outcome measure in trials or surveillance measuring the incidence of *P. falciparum* malaria, such as our study. A longitudinal study conducted over 69 weeks in Papua New Guinea on children aged 0.9 to 3.2 years found higher FOI values. Our lower values may be explained by the fact that in our study, samples were collected outside the main malaria transmission season. Collection between the months of June and September (peak of rainy season) would probably have shown higher values. An increase in FOI with age was observed, confirming previous entomological data showing that the number of infectious bites increased with body size [44] and that FOI increased with age [45]. Our results could be explained by the fact that malaria vectors are known to bite easily outdoors and in the early hours of the night, so older children, being more independent (and possibly unprotected), may be at greater risk of being bitten outdoors. Indeed, in a previous study conducted in Banfora, the results indicated that vector bites in the early evening were observed in our study area and that this was the period during which it was common to find active communities in the peri-domestic environment [46]. This increase in FOI with age in our study could alter assumptions that children under five are the most infected and raise the question of reorienting the target audience for SMC coverage.

In this study, older children appear to be more exposed to clinical cases of malaria. A study with opposite results showed that repeated exposure to *P. falciparum* infections in previous years could confer immunity. In this study, cases of *P. falciparum* malaria observed in a population exposed to a high level of transmission showed that the acquisition of antimalarial immunity with age was accompanied by a reduction in the duration and frequency [47].

The limitation of this study could be that the genetic diversity was incorrectly estimated due to the detection limit of the PCR technique. Thus, our results could be explained by a possible underestimation of the exact number of alleles due to limitations of the analytical techniques used in genotyping in contrast to other studies, such as the one conducted by Ibrar Ullah et al., because it is difficult to distinguish fragments with length differences less than 20 bp [48] or with the use of the amplicon sequencing [49,50]. In addition, this study was not designed to answer a question related to alleles.

## 5. Conclusions

Multiple infections were reported in the study subjects, the majority of whom were aged 1.5 to 12 years. Older age and the MOI appear to be protective factors against the occurrence of clinical malaria episodes. The polyclonality and rainy months (coinciding with the high transmission season) are predictive factors associated with MOI and with the acquisition of new clones. The results show that FOI and MOI are age dependent, with the youngest children being more affected. This justifies the current malaria control strategies targeting children under five years of age. Extending the control strategy to children above five years of age will probably lead to better control of malaria in endemic countries.

## Figures and Tables

**Table 1:** Characteristics of the samples.

Characteristic	*n* (%)
Age	
˂5years	191 (41.16)
≥5years	273 (58.84)
Sex	
Male	229 (49.35)
Female	235 (50.65)
Protection tools against mosquitoes’ bites	
ITN	94 (20.30)
Insecticide only	3 (0.65)
ITN + Insecticide	358 (77.32)
None	8 (1.73)

**Table 2:** Predictors of time to onset of clinical episodes.

	Unadjusted Model			Adjusted Model		
	IRR	95% CI	*p* Value	IRR	95% CI	*p* Value
Age	0.78	[0.67–0.91]	0.002	0.74	[0.61–0.90]	0.002
Gender	0.74	[0.33–1.69]	0.482	0.41	[0.16–1.08]	0.997
Polyclonality	0.75	[0.18–3.08]	0.687	0.49	[0.11–2.22]	0.354
MOI	1.30	[1.03–1.64]	0.025	1.43	[1.04–1.99]	0.029
FOI	-	-	-	0.99	[0.70–1.43]	0.997
Log likelihood	−90.85			−66.43		
AIC	191.69			144.85		

95% CI, 95% confidence interval; IRR, incidence rate ratio; AIC, Akaike information criterion.

**Table 3:** Predictive factors for MOI.

	Univariate Analysis			Multivariate Analysis			Adjusted Model		
	IRR	95% CI	*p* Value	IRR	95% CI	*p* Value	IRR	95% CI	*p* Value
Age Group									
˂5years	1 (base)			1 (base)					
≥5years	0.87	[0.75–0.99]	0.042	0.87	[0.73–1.046]	0.144			
Gender									
Male	1 (base)			1 (base)					
Female	1.08	[0.96–1.22]	0.217	1.026	[0.88–1.19]	0.719			
Polyclonality									
No	1 (base)						1 (base)		
Yes	4.001	[3.10–5.15]	˂0.001	2.90	[2.13–3.96]	˂0.001	2.962	[2.17–4.03]	˂0.001
Treated (treatment administrated during follow-up)									
No	1 (base)			1 (base)					
Yes	0.69	[0.61–0.78]	˂0.001	0.88	[0.73–1.08]	0.504			
Fever									
No	1 (base)			1 (base)					
Yes	1.34	[1.13–1.59]	0.001	1.23	[0.97–1.54]	0.084			
Study site									
Bounouna	1 (base)			1 (base)					
Nafona	1.24	[1.10–1.40]	˂0.001	1.12	[0.95–1.31]	0.172			
FOI	1.27	[1.03–1.64]	˂0.001	1.17	[1.12–1.23]	˂0.001	1.168	[1.12–1.22]	˂0.001
Parasitemia	1.46	[1.28–1.66]	˂0.001	1.17	[0.99–1.39]	0.072	1.239	[1.06–1.45]	0.006
Season of sample collection									
Dry months	1 (base)			1 (base)			1 (base)		
Rainy months	1.51	[1.32–1.72]	˂0.001	1.62	[1.31–1.99]	˂0.001	1.680	[1.38–2.04]	˂0.001

95% CI, 95% confidence interval; IRR, incidence rate ratio.

**Table 4:** Predictive factors for FOI.

	Univariate Analysis			Multivariate Analysis			Adjusted Model		
	IRR	95% CI	*p* Value	IRR	95% CI	*p* Value	IRR	95% CI	*p* Value
Age Group									
˂5years	1 (base)			1 (base)					
≥5years	0.80	[0.66–0.98]	0.032	0.81	[0.65–1.00]	0.060			
Gender									
Male	1 (base)			1 (base)					
Female	1.12	[0.94–1.34]	0.187	1.09	[0.91–1.31]	0.327			
Polyclonality									
No	1 (base)			1 (base)			1 (base)		
Yes	4.98	[3.36–7.39]	˂0.001	2.47	[1.62–3.77]	˂0.001	2.46	[1.62–3.76]	˂0.001
Treated (treatment administrated during follow-up)									
No	1 (base)			1 (base)					
Yes	1.45	[1.20–1.76]	˂0.001	1.07	[0.85–1.36]	0.542			
Fever									
No	1 (base)			1 (base)					
Yes	1.45	[1.13–1.84]	0.003	0.89	[0.66–1.19]	0.435			
Study site									
Bounouna	1 (base)			1 (base)					
Nafona	1.31	[1.10–1.55]	0.002	1.13	[0.92–1.39]	0.235			
MOI	1.0004	[0.92–1.08]	0.993	1.26	[1.20–1.33]	˂0.001	1.27	[1.22–1.34]	˂0.001
Parasitemia	1.55	[1.28–1.86]	˂0.001	1.07	[0.86–1.33]	0.530			
Season of sample collection									
Dry months	1 (base)			1 (base)					
Rainy months	2.08	[1.65–2.61]	˂0.001	2.02	[1.57–2.60]	˂0.001	2.09	[1.67–2.63]	˂0.001

95% CI, 95% confidence interval; IRR, incidence rate ratio.

## Data Availability

The original contributions presented in the study are included in the article, further inquiries can be directed to the corresponding author.
